# A Robust Indoor Positioning Method based on Bluetooth Low Energy with Separate Channel Information

**DOI:** 10.3390/s19163487

**Published:** 2019-08-09

**Authors:** Baichuan Huang, Jingbin Liu, Wei Sun, Fan Yang

**Affiliations:** 1 State Key Laboratory of Information Engineering in Surveying, Mapping and Remote Sensing, Wuhan University, Wuhan 430079, China; 2 Department of Remote Sensing and Photogrammetry and the Center of Excellence in Laser Scanning Research, Finnish Geospatial Research Institute, 02430 Masala, Finland; 3 Key Laboratory of Precise Engineering and Industry Surveying, National Administration of Surveying, Mapping and Geoinformation, Wuhan University, Wuhan 430079, China; 4 Wuhan Geomatics Institute, Wuhan 430079, China; 5 State Key Laboratory of Geodesy and Earth’s Geodynamics, Chinese Academy of Sciences, Wuhan 430079, China

**Keywords:** indoor positioning, Bluetooth low energy (BLE), separate channels, separate signal-attenuation models, distance decision strategy, weighted trilateration

## Abstract

Among the current indoor positioning technologies, Bluetooth low energy (BLE) has gained increasing attention. In particular, the traditional distance estimation derived from aggregate RSS and signal-attenuation models is generally unstable because of the complicated interference in indoor environments. To improve the adaptability and robustness of the BLE positioning system, we propose making full use of the three separate channels of BLE instead of their combination, which has generally been used before. In the first step, three signal-attenuation models are separately established for each BLE advertising channel in the offline phase, and a more stable distance in the online phase can be acquired by assembling measurements from all three channels with the distance decision strategy. Subsequently, a weighted trilateration method with uncertainties related to the distances derived in the first step is proposed to determine the user’s optimal position. The test results demonstrate that our proposed algorithm for determining the distance error achieves a value of less than 2.2 m at 90%, while for the positioning error, it achieves a value of less than 2.4 m at 90%. Compared with the traditional methods, the positioning error of our method is reduced by 33% to 38% for different smartphones and scenarios.

## 1. Introduction

Positioning, navigation, timing, remote sensing and communication (PNTRC) systems will change our life greatly in the future [1]. In the PNTRC system, the most fundamental and important technologies are seamless positioning and ubiquitous navigation. In fact, outdoor positioning the technology is relatively mature, owing to satellite positioning systems like Global Navigation Satellite System (GNSS). However, currently, there is no single indoor positioning technology that is able to balance cost, accuracy, performance, robustness, complexity, and limitations [2,3,4]. The general indoor positioning technologies include infrared positioning [3,5], ultrasound positioning [3,6], radio frequency positioning [4], magnetic positioning [7], microelectromechanical systems positioning [8,9], vision-based positioning [10] and audible sound positioning [11,12]. In particular, radio positioning technologies, such as radio frequency identification (RFID), wireless LAN (WLAN), ZigBee, Bluetooth low energy (BLE) and ultrawideband (UWB), have drawn much attention because of the issuance of many wireless radio standards [2,3]. Among these wireless systems, the most commonly used ones for indoor positioning are WiFi and Bluetooth [13].

WiFi has over 50 sub-bands on the 2.4 GHz band, and each of them has a bandwidth of 20 MHz. Most of the past pioneering work simply used the WiFi received signal strength indication (RSSI), which reflects the aggregate value of all channel information [14]. The reason why they did not take advantage of the sub-band information is that the WiFi Access Point (AP) used to collect information such as RSSI, but RSSI was originally designed for communication so that the sub-band information remains private for users. The use of RSSI in previous works [4,13,15,16,17] has suffered from many problems, such as body blockage, the temporal environmental change effect, etc. Unlike the RSSI, the recent studies leveraging channel state information (CSI) from all sub-bands have revealed that CSI has more fine-grained information than RSSI; hence, CSI is more robust against body blockage and the temporal environmental change effect, and therefore, a better positioning performance can be expected [16,18,19].

**Figure 1:**
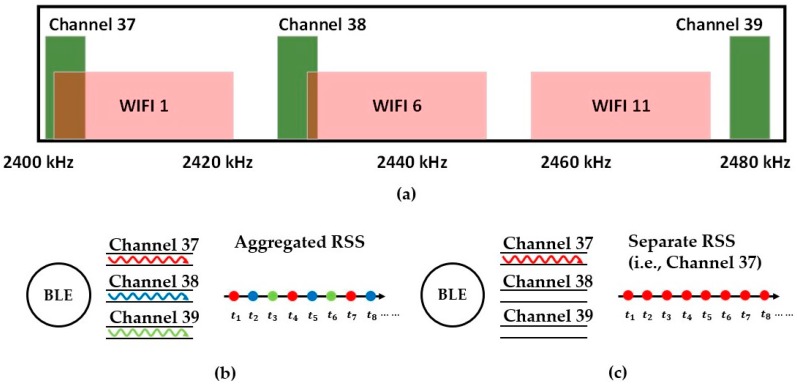
(**a**) The three advertising channels of BLE and the three most common WiFi sub-bands. Channels 37, 38 and 39 are the advertising channels of BLE. The workflow of (**b**) aggregated RSS and (**c**) Separate RSS (i.e., Channel 37).

The concept of sub-bands can also be employed in BLE positioning. Similarly, BLE has 40 channels on the 2.4 GHz band, and each channel has a bandwidth of 2 MHz [14]. In addition, the BLE has become the focus of current indoor positioning technologies because it takes advantage of low power consumption and easy deployment [20]. A comparison of the frequency bands between WiFi and BLE is illustrated in Figure 1a. Channels 37, 38 and 39 of BLE are advertising channels, broadcasting messages in loops, and the other 36 channels are designed for communication in connection [21]. Meanwhile, the aggregate received signal strength (RSS) is sampled in the loop of three advertising channels [14], see Figure 1b,c. In fact, the different channels will produce the distinguished RSS measures because of the frequency band the channel occupies. Therefore, an aggregate RSS loses the fine-grained information and will diminish the details of the RSS measure from each channel. To this end, we propose taking advantage of the three separate channels of the BLE, similar to the CSI approach that has been successfully applied for WiFi positioning systems.

To date, BLE-based indoor positioning technologies can be classified into a geometric mapping approach and fingerprinting approach, with the former being the primary focus of this study [16]. In this case, methods such as triangulation, trilateration, and multilateration are generally used to locate the target [22]. The aim of these methods is to accurately measure the distance between the target and APs by leveraging the time of arrival (TOA), time difference of arrival (TDOA), RSS, time of flight (TOF) and received signal phase [4,16]. Regarding only the RSS approach, the distance can be calculated by using the instant RSS measure and a radio-propagation model that was established in advance. However, the distance estimation with the prebuilt radio propagation model in a complicated indoor environment suffers from many problems, for instance, NLOS (not line of sight) [23], the multipath effect and path loss [14], signal reflection [22], moving objects [4,24], a noise floor [25], and 802.11 interference [25], which are summarized as spatial variations. In detail, the so-called 802.11 interference of BLE denotes the effect caused by WiFi because WiFi and BLE run on the same band (2.4 GHz); see Figure 1a [14,21,25]. In addition, the narrow bandwidth of BLE advertising channels makes individual RSS tend to fade fast, and the protocol of receiving an advertising package makes the aggregate RSS tend to fluctuate [20]. Because of the interference depicted before, the traditional RSS measurement (aggregate RSS from three channels) usually presents a larger uncertainty, which leads to an incorrect distance estimate with the prebuilt single radio propagation model.

In this paper, we aim to achieve a more robust and precise indoor positioning system. The structure of the paper is as follows: Section 2 presents the previous work and the background of the indoor positioning of BLE. In Section 3, the algorithms contain a stable RSS inquire, the separate signal-attenuation models with the strategy of distance decision and weighted trilateration. Separate channels and signal-attenuation models are designed to obtain a more stable RSS. In the meantime, the distance decision and weighted trilateration strategies are used to increase the precision and adaptability. Section 4 demonstrates the experimental setup and performance of the algorithms. Finally, Section 5 presents the conclusions and future directions of research.

## 2. Related Work

To obtain a more stable RSS, Kim [26] proposed an accurate indoor proximity zone detection technique based on the time window and frequency of the RSS. Ozer [27] improved the performance of BLE indoor positioning using the Kalman filter to make the RSS smoother. However, he could not avoid the use of dirty data, resulting in a bad result. Furthermore, Ishida [28] first proposed BLE separate channel fingerprinting using channel-specific features. Jovan [21] and Ramsey [14] used three advertising channels to build fingerprinting maps in the process of removing fast fades with an iPhone to investigate the value of separate channel information. Moreover, Giovanelli [29] used the CC2650 tag and nRF52840 to estimate the distance covered by the signal corresponding to the RSS. Nikoukar [25] measured the noise floor in different channels using nRF52480 SoC in different environments to mitigate the noise and measured the separate RSS values. Although these authors tried to simulate the real environment, they obtained only separate channel information with specific devices. In this paper, we focus on the need for easy deployment. Beacons are arranged in separate channels so that an off-the-shelf smartphone can work in our system.

To improve the precision and adaptability, Subha [30] and Ali [25] proposed adding particular terms to the original model to absorb the multipath error. Yu [31] put forward a new BLE propagation model according to the location of the BLE beacons and fused the propagation model and multiple sensors through the Kalman filter. Moreover, Canton Paterna [32] used frequency diversity, the Kalman filter and a trilateration method to improve the precision of the BLE system. He compared the largest RSS value, the mean RSS value and the maximum ratio combined with separate RSS (channel 37, 38 or 39) to choose the best RSS value. He also compared the log-distance path loss model with shadowing, the International Telecommunication Union (ITU) model for indoor environments and the empirical model to choose the best model for indoor location in the large analysis. However, Canton Paterna did not improve the model to simulate the real environment. He chose only the best channel to estimate the most accurate position without considering the changeable space and time variations in indoor environments. Zhuang [33] combined the channel-separate polynomial regression model, channel-separate fingerprinting, outlier detection and the extended Kalman filter. He experimented in densely and sparsely distributed BLE beacons in all three advertising channels to fit the best model and to generate the fingerprinting without considering the bad channel results. However, these authors ignored the fact that indoor environments are changeable and complicated. In contrast, we combine the separated channels and the distance decision strategy to improve the adaptability. In particular, separate signal-attenuation models provide the estimate distance for the distance decision strategy. The distance decision strategy is directly applied to every channel and evaluates the comparison of each. Moreover, weighted trilateration with the estimate distance derived from the distance decision strategy performs well in obtaining the optimal position.

## 3. Algorithm

### 3.1. Separation of the BLE Channels for a More Stable Signal

**Figure 2:**
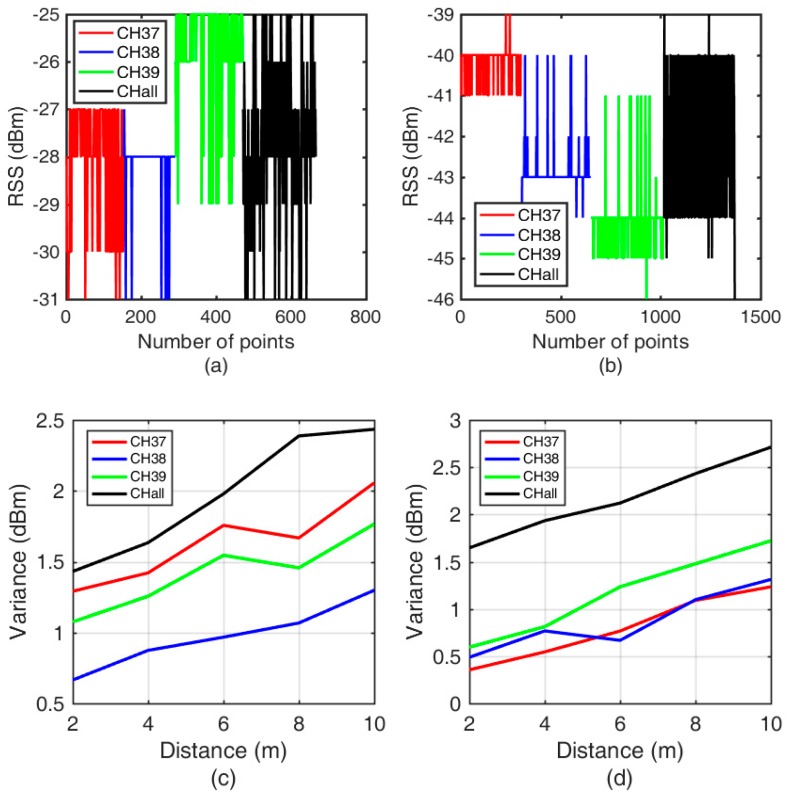
Comparisons between separate RSS values and the aggregate RSS when using the (**a**) Google Pixel 3 L and (**b**) Huawei P20 (for a distance of 2 meters). The variance of the separate RSS values and the aggregate RSS with increasing distance using the (**c**) Google Pixel 3 L and (**d**) Huawei P20.

BLE broadcasts the messages in quick succession. In the protocol layer, CH 37 (channel 37), CH 38 (channel 38), and CH 39 (channel 39) broadcast the same message repeatedly to avoid package loss. Regarding the listening rule, devices such as smartphones listen to broadcasted messages circulating in CH 37, CH 38 and CH 39. However, the final RSS obtained from BLE is indeed CH all (the aggregated RSS from all channels), which is summed from the three separate channels in loops. According to the rule of error propagation, the aggregated RSS has a larger uncertainty than that of each channel. Acquiring a more stable RSS, deemed the foundation of positioning, requires separate channels. Fortunately, separate channel can’t keep in bad and stable character all the time [21]. In this design, three beacons are scheduled to run separately in CH 37, CH 38 and CH 39, while the other beacon is a normal beacon that broadcasts the aggregated RSS as a benchmark. A simple experiment was carried out between two brands of mobile phones to demonstrate the behaviors of individual RSS and the aggregated RSS (see Figure 2).

It can be seen from Figure 2a that the RSS of CH 37 ranges from −31 dBm to −27 dBm. In addition, the RSS ranges from −31 dBm to −28 dBm for CH 38 and from −29 dBm to −25 dBm for CH 39. The variance of CH all (1.6756) is higher than one of CH 37 (0.9915), CH 38 (0.6654) and CH 39 (0.7756). The results imply that the RSS varies greatly with the channel, which is also supported by Figure 2b. Furthermore, it can be seen that the aggregate RSS ranges from −31 dBm to −25 dBm. The aggregate RSS apparently has a larger variance than that of each channel, at almost all distances (see Figure 2c,d). The variance of CH all (1.6465) is higher than one of CH 37 (0.3555), CH 38 (0.4885) and CH 39 (0.5952). The results are consistent with our previous assertation that an individual RSS should be more stable. Therefore, with the challenge of temporal variations, separate channels instead of an aggregate one are adopted in this study to acquire a more stable RSS measurement and to improve the positioning performance because of the stability of data source.

### 3.2. The Distance Decision Strategy

**Figure 3:**
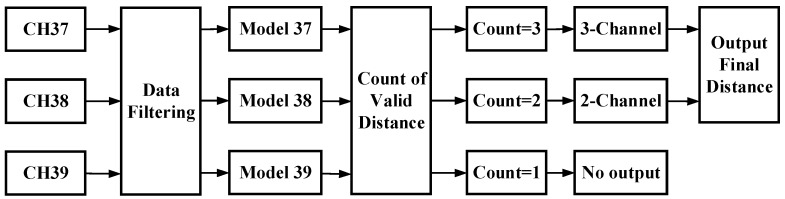
Overview of the proposed algorithm for outputting the final distance.

As separate channels are used in this study, a distance decision strategy regarding the distance between the receivers (smartphones) and senders (BLE beacons) should be developed to fully exploit all the channel information. Here, three steps are taken to achieve the objective; see the workflow shown in Figure 3. In the first step, three separate signal-attenuation models are trained in the offline phase. It’s necessary but not complex. Second, data filtering is implemented to eliminate abnormal RSS points (outliers) and interpolate the fixed point. Finally, a method is proposed for fusing the separate distances estimated for each channel and outputting the final distance.

#### 3.2.1. Separate Signal-Attenuation Models in the Offline Phase

The distance can be estimated by measuring the instant RSS and using a prebuilt radio propagation model that reveals the one-to-one corresponding relationship between distance and RSS. Actually, the log-distance path loss model was most frequently used in previous works because the channel fading characteristic contains a log-normal distribution [23,32,33]. Therefore, our study chooses this model as well to train the signal-attention models, as shown below:

(1) $$RSS=RSS\left(d_{ref}\right)+10\alpha \log\left(\frac{d}{d_{ref}}\right)+X_{\sigma }$$

where RSS is a dependent variable, d is an independent variable, $d_{ref}$ is the reference distance, and $RSS\left(d_{ref}\right)$ is the RSS at the reference point. α is the parameter representing the path-loss exponent, and $X_{\sigma }$ follows a Gaussian distribution, with zero mean [33].

It is apparent that three sets of signal-attenuation models are needed because the information of three BLE channels is utilized in our design. Therefore, we establish three signal-attenuation models ($\mathrm{denoted}\;\mathrm{as}\;M_{37}$,$\;M_{38}$ and $M_{39}$) with respect to CH 37, CH 38 and CH 39, respectively, in the offline training phase. However, only one model was trained for the aggregated RSS in the conventional method. It is apparent that three separate models can better reveal the feature of each channel information. This offline training phase normally includes two steps: (1) collection of the RSS during a given time period at a fixed position, and use of the mean value of the RSS measurements as the “observation” of this position; (2) the recording of a set of “observations” and the distances between the receivers (smartphones) and senders (BLE beacons). In this way, a least-square method can be utilized to find the optimal value of parameter α in Equation (1) in the offline phase. Under the framework of the least-square method, the three parameters can be obtained. $\alpha_{37}$ represents the path-loss exponent in CH 37. CH 38 and CH 39 each may have a different parameter as $\alpha_{38}$ or $\alpha_{39}$.

**Figure 4:**
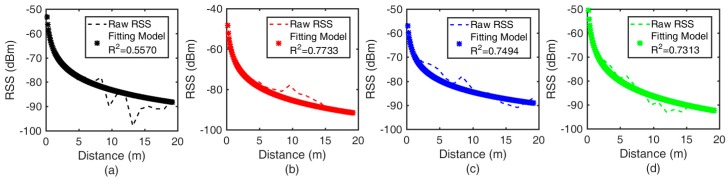
The signal-attenuation models for the Google Pixel 3 L: the fitting model for (**a**) CH all: $R^{2}$ = 0.5570; (**b**) CH 37:$\;R^{2}$ = 0.7733; (**c**) CH 38:$\;R^{2}$ = 0.7494; and (**d**) CH 39:$\;R^{2}$ = 0.7313.

To test the separate signal-attenuation models, a long corridor is selected as our experimental environment, with the Google Pixel 3 L being used. Sixteen positions are chosen, and the distance between each pair of adjacent positions is 1.2 m. Collection of the RSS for 5 minutes at every fixed position to a distance of 19.2 m is carried out, and the mean value of the collection is regarded as the raw RSS. The raw RSS values are recorded with the true distance at every fixed position. In the offline phase, the record is trained to fit the signal-attenuation models (see Figure 4). The dotted line represents the mean value of the collected RSS at every fixed position, and the symbol ’*’ represents the signal-attenuation models. $R^{2}$, called the goodness of fit, is employed to evaluate the established model. It is apparent from Figure 4 that the separate signal-attenuation models have a higher $R^{2}$ values than those of the aggregate signal-attenuation models. In the offline phase, the separate signal-attenuation models represent the relatively optimal models.

In the online phase, the distance should then be estimated from each signal-attenuation model ($M_{37}$, $M_{38}$ and $M_{39}$). To verify the separate models in practice, another test in the same long corridor using the Google Pixel 3 L is carried out. In this phase, the RSS is collected for 10 s at every fixed point, as in the offline phase. Every received RSS is input into the separate signal-attenuation models to obtain an estimate distance. By recording the true distance and the estimated distance inferred from the models, we can output the CDF (cumulative distribution function) map of the errors, as shown in Table 1.

The implication of the results shown in Table 1 is that all three signal-attenuation models can estimate a distance from the smartphones to the beacons. The 90 percentile distance errors are no less than 4.0 m, which is not sufficient for precise indoor positioning. For this, a distance decision algorithm with data filtering is proposed to achieve adaptable and robust indoor positioning.

#### 3.2.2. Data Filtering

In this study, three beacons (each of them is coded in the level of firmware to broadcast only one channel) are combined as a station to achieve the broadcast of separate channel information. It is worth mentioning that some of the past works directly modified the hardware of a single beacon to achieve the broadcast of three channels of information at the same time. Unlike previous works, we bypass the technical difficulty of modifying the hardware and manage to obtain the three channels of information by simply combining three beacons as a station (each of them is coded in the level of firmware to broadcast only one channel). Despite this convenience, our method may introduce a new problem, that is, the packet loss of some channels during one round of a scanning. We have assumed that the RSSs from the three channels are completely synchronized, which is contrary to reality. Hence, we develop a “lost detection” method to tag the channel that is unavailable for one round of a scanning. In addition, outliers that make great jumps should be detected and removed/smoothed at this stage, which can be achieved by the median filter algorithm [34]. The details are expanded in the following.

During a given time period, the received package has a different number of detected beacons. If one beacon is not detected, the RSS of this beacon in this round of scanning is an empty one, that is, it is lost. The “lost” RSS is critical for the later distance decision, so we have to distinguish which beacon is “lost”. To this end, we carry out loss detection as follows (see Equation (2)), which is actually a process of tagging. When $R_{i}$ is empty, $T_{i}$ is equal to 0. In contrast, $T_{i}$ is equal to 1 if $R_{i}$ is not empty. Loss detection provides the tags needed for median filtering:

(2) $$T_{i}=\{\begin{array}{c} 0\;\;\;\;\;R_{i}\;is\;empty\;\;\;\;\;\;\;\\ 1\;\;\;\;\;R_{i}\;is\;not\;empty \end{array}$$

where $T_{i}$ is the tag in time period i and $R_{i}$ represents the received RSS in time period i.

After loss detection, median filtering is carried out to address the data with tags sequentially. Here, we take CH 37 as an example to illustrate the process of median filtering. $\left\{RSS_{37,1},RSS_{37,2},RSS_{37,3},\;\ldots ,\;RSS_{37,i}\right\}$, which denotes the RSS sequence of CH 37 (different smartphones have different sampling frequencies) during the sampling period, has a matched tag sequence $\left\{tag_{37,1},tag_{37,2},tag_{37,3},\;\ldots ,\;tag_{37,i}\right\}$, found by Equation (2). A sliding window of $WL_{1}$ in length is adopted to sample the median value. For $i=1,2\ldots ,\;t-WL_{1}+1$, we have:

(3) $$RF_{i}=\{\begin{array}{ll} R_{i},R_{i+1},\ldots R_{i+WL_{1}+1} & T_{i}=1\;\\ None & T_{i}=0 \end{array}\;$$

where $WL_{1}$ is the length of the sliding window, $RF_{i}$ is the selected RSS and $R_{i}$ represents the RSS of CH 37 in time period i; when the total count is t, the max of i is $t-WL_{1}+1$.

In other words, the purpose of median filtering is to remove the outlier, which behaves as a sudden jumping point. Mainly, $T_{i}$, the tag of the last point in the sliding window, is deemed the decisive point. If the value of the decisive point is zero, the whole sliding window should be thrown out. Correspondingly, some fixed points, the median values of the sliding window, should be inserted.

**Figure 5:**
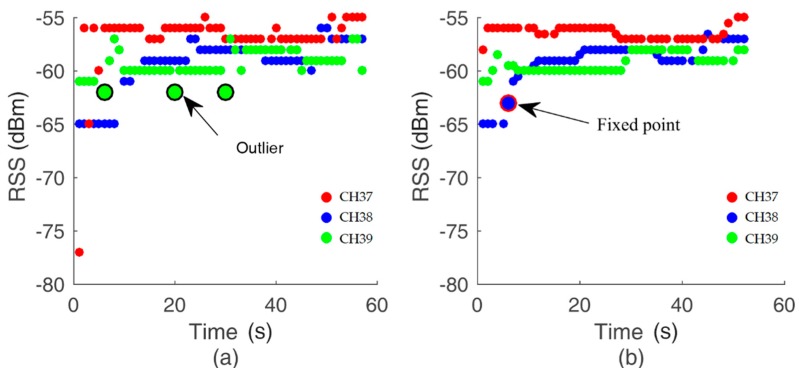
The scatter plot of (**a**) raw data; (**b**) processed data. Here, $WL_{1}\;$ is 5 in Equation (3).

To test the approach of median filtering and loss detection, in the same long corridor, RSS values are collected at every fixed position for 10 s, as in the online phase. For example, the set of RSS values for the Huawei P20 at a fixed position 3 m away before and after the methodology is shown in Figure 5. When the value of $WL_{1}\;$is 5, the probability of the outlier being here is 15%. In fact, the probability of outliers mainly depends on the environment, which we cannot improve. Figure 5a shows the plot of raw data, while the reprocessed data are shown in Figure 5b. The number of outliers in Figure 5a has been considerably reduced in Figure 5b. After median filtering, the sharp noise was deleted fully. Meanwhile, the process of loss detection is shown in Figure 5b. The fixed point has been inserted. On the whole, the process of data filtering makes the RSS values more gathered and smooth. The combination of loss detection and median filtering can output a reprocessed and clean sequence of RSSs for later use in the distance decision strategy.

#### 3.2.3. Distance Decision

After data filtering, the three sets of RSS values correspond with a series of distances in separate channels. For example, the $i_{\mathrm{th}}$ point of the filtered RSS in CH 37 can output a distance result called $D_{37}$, as well as $D_{38}$ and $D_{39}$. However, it should be noted that one can never expect to acquire $D_{37}$, $D_{38}$ and $D_{39}$ every time, as some of these parameters might be lost, as depicted before. To synthesize the final distance, the distance decision algorithm is implemented to analyze and optimally combine the three estimated distances derived from the separate signal-attenuation models. With the hypothesis that three beacons in three channels can be used to output the final distance, our distance decision strategy is illustrated as follows according to the number of valid distances.

2-Channel is used when the number of valid distances (valid channel information) is two. In this case, the possible combinations are presumably $D_{37}$ and $D_{38}$, $D_{37}$ and $D_{39}$, or $D_{38}$ and $D_{39}$. Two steps are followed: (1) detecting the variance of a single channel and (2) detecting the difference between the two channels.

For instance, $D_{Q}$ and $D_{P}$ (Q,P = 37, 38, 39) are valid here. In the first step, there is a threshold and a sliding window for variance detection. The variance detection requires that $\sigma <Thr_{1\;\;\;}$(where σ is the variance of a single channel and $Thr_{1}$ represents the threshold of variance in step one). Only if the two specific channels meet the requirement of variance detection does step two, called difference detection, commence. The process of difference detection in 2-Channel can be described mathematically as shown in Equation (4):

(4) $$\frac{\sum_{i=1}^{i+WL_{2}-1}|D_{Q,i}-D_{P,i}|}{WL_{2}}<Thr_{2}$$

where $WL_{2}$ is the length of the sliding window, $Thr_{2}$ represents the threshold of variance in difference detection in time period i, $\;D_{Q,i}$ is the value of the distance in channel Q and $D_{P,i}$ is the value of the distance in channel P.

As a consequence, the ultimate output distance $D_{i}$ is the weighted average of $D_{Q}$ and $D_{P}$:

(5) $$\{\begin{array}{c} D_{i}=m_{1}*D_{Q,i}+m_{2}*D_{P,i}\\ m_{1}=\frac{\sigma_{P}}{\sigma_{Q}+\sigma_{P}}\\ m_{2}=\frac{\sigma_{Q}}{\sigma_{Q}+\sigma_{P}} \end{array}$$

where $m_{1}$ and $m_{2}$ are the weights for channel Q and channel P, respectively, $\sigma_{Q}$ is the variance of channel Q, and $\sigma_{P}$ is the variance of channel P. High variance in variance detection means more interference and lower weight.

3-Channel is used when the number of valid distances is three. In addition, 3-Channel is more precise and persuasive than 2-Channel in practice because of the greater availability of measurements. Similarly, two steps are also followed: (1) detecting the variance of each of the three channels separately and (2) detecting the difference between every two channels. The differences between any two of the three distances ($D_{37}$, $D_{38}$, and $D_{39}$) should be taken into account.

In the first step, there is a threshold and a sliding window for variance detection. The variance detection requires that $\sigma <Thr_{1}$, as demanded by 2-Channel (where σ is the variance of a single channel and $Thr_{1\;\;\;}$ represents the threshold of variance in step one). Only if all three separate channels fulfill the requirement of variance detection is step two, called difference detection, commenced. The process of difference detection in 3-Channel can be described mathematically as shown in Equation (6):

(6) $$\{\begin{array}{c} \frac{\sum_{i=1}^{i+WL_{3}-1}|D_{37,i}-D_{38,i}|}{WL_{3}}<Thr_{3}\\ \frac{\sum_{i=1}^{i+WL_{3}-1}|D_{37,i}-D_{39,i}|}{WL_{3}}<Thr_{3}\\ \frac{\sum_{i=1}^{i+WL_{3}-1}|D_{38,i}-D_{39,i}|}{WL_{3}}<Thr_{3} \end{array}$$

where $WL_{3}$ is the length of the sliding window, $Thr_{3}$ represents the threshold of variance in difference detection in time period i, $D_{37,i}$ is the value of the distance in CH 37, $D_{38,i}$ is the value of the distance in CH 38 and $D_{39,i}$ is the value of the distance in CH 39.

Accordingly, the ultimate output distance $D_{i}$ is the weighted average of distance 37, distance 38 and distance 39:

(7) $$\{\begin{array}{c} D_{i}=m_{1}*D_{37,i}+m_{2}*D_{38,i}+m_{3}*D_{39,i}\\ m_{1}=\frac{\sigma_{38}+\sigma_{39}}{2*(\sigma_{37}+\sigma_{38}+\sigma_{39})}\\ m_{2}=\frac{\sigma_{37}+\sigma_{38}}{2*(\sigma_{37}+\sigma_{38}+\sigma_{39})}\\ m_{3}=\frac{\sigma_{37}+\sigma_{38}}{2*\left(\sigma_{37}+\sigma_{38}+\sigma_{39}\right)} \end{array}$$

where $m_{1}$, $m_{2}$ and $m_{3}$ are the weights for CH 37, CH 38 and CH 39, respectively, $\sigma_{37}$ is the variance of CH 37, $\sigma_{38}$ is the variance of CH 38 and $\sigma_{39}$ is the variance of CH 39.

In conclusion, the quality of broadcasting signal is affected by many reasons like walking people, fixed obstacle (desk, chair, cupboard…) and state of transmitter/receiver, etc. Because of these complicate factors in indoor environment, the quality of broadcasting is varying time to time, and there should be thus no fixed relation between the quality and the real distance. However, a direct quality evaluation is possible in our method, by step (1) detecting the variance of each channel separately and step (2) detecting the difference between every two channels. In this way, our method only employs the RSS of “good quality”, resulting in a better distance estimation in theory.

The basic rationale behind our distance decision strategy is that: the interruptions caused by unpredictable factor like walking people on the three channels are different. In another word, the three channels will consistently reflect a same distance estimate if no interruption, however, a large interruption will lead to a large discrepancy of distance estimates between the three channels. By detecting how large is the discrepancy between channels, our distance decision strategy can tell if this is a good or bad measure, and avoid to output result in bad quality. However, the traditional method will adopt all channel outputs without any particular treatment with the error.

**Figure 6:**
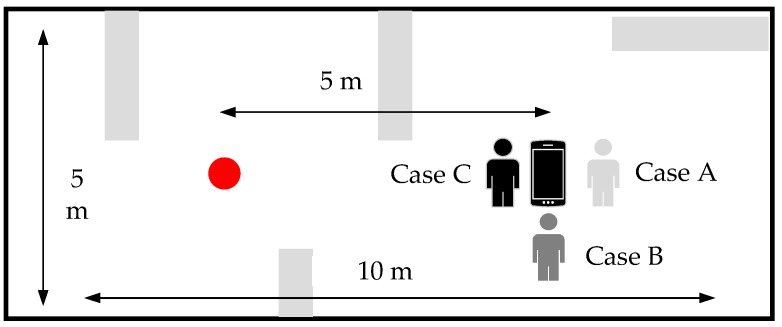
Test setup: the red point is the station; the gray bars denote desks and cupboards; and the different positions of people represent different cases.

In particular, a validation is set up to test our distance decision strategy, as Figure 6 illustrates. The distance between the station and the smartphone (Google Pixel 3 L) is 5 m. The test is set up as real as possible by (1) distributing some desks and cupboards as obstacles and (2) rotating the smartphones. Although the real case is usually more complicated than our setup, we suggest that the experimental results under our setup still make sense. In addition, three locations (A, B, C) are tested in the experiment: case A in Figure 6 represents the status of inherent influence. The case of a smartphone backing away from the station is marked as case C (blocked). In this case, the line of sight is blocked. The case in which a smartphone is positioned vertically with straight lines between the station and smartphones is considered case B (not blocked). The three cases provide powerful evidence that the direction of the smartphone makes a difference. Table 2 shows the results of the different cases when $Thr_{1}$ is 3 $m^{2}$ in the first step of the distance decision strategy, and $Thr_{2}$ in Equation (4), $Thr_{3}$ in Equation (6) are both 5 m.

In fact, NLOS is one of the main factors that may deteriorate distance estimation from RF signals (WiFi/BLE), and many papers in previous dedicate to minimize the NLOS effect [35], while none of them claims to completely solve the NLOS problem as far as we know. Unlike the previous work that try to calibrate the RSS measures even in NLOS case, this study prefers to avoid result output (usually in bad quality) in NLOS case (or other cases, not limited to NLOS) so as to stabilize the positioning. As Table 2 demonstrates, in this trial, no results are output when the line of sight is blocked in case C. Because of the tails of the separate signal-attenuation models, a small jitter will result in a large change. When the line of sight is blocked, the value of the RSS becomes very small, which will cause a large error in terms of the distance. Hence, the case will not meet the requirement of the distance decision. In other words, the probability of no output in case C is 100%. In case B, the direction of the smartphones does not improve stability compared with that in case A, being marked as an inherent influence. Thus, the number of times no output occurs in case B is higher than that in case A. The rationale behind our distance decision strategy is that a more precise distance can be obtained with more consistent channel information among the three advertising channels. In brief, the distance result is convincing when the three channels output similar distances.

By combining the use of separate channel information and the distance decision strategy, we suggest that the result should have more adaptability and more robustness to signal interruption caused by, for example, body blockage. Our distance decision strategy based on a relatively long sequence can achieve greater positioning accuracy, which is the major objective of this study.

### 3.3. Weighted Trilateration

With the distances measured from several stations, a weighted trilateration method is proposed to realize positioning. In our experimental setup, a location station is equipped with three beacons, and each beacon is arranged such that it has one advertising channel, as described before. Considering the occurrence of packet loss at our location station, a four-station setup is suggested, which is also very popular among the state-of-the-art geometric positioning systems.

Trilateration can succeed in locating the target when the measurements taken converge at a single point. However, in a practical environment, there are some distance errors that lead to an area of possible locations instead of a single location point. Derivation from the difference detection of each station in Section 3.2.2 is utilized to increase confidence regarding the receivers (smartphones) closer to the senders (stations) by moving the target point.

**Figure 7:**
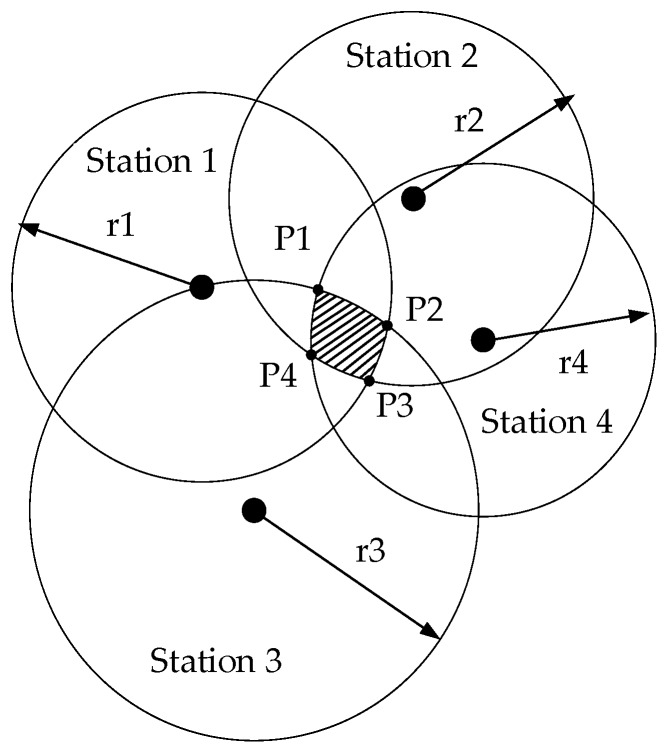
Four intersecting circles with evaluated errors.

In the case of evaluated errors shown in Figure 7, the intersection of circles creates an area. The maximum likelihood estimate, the least-square method or the triangle centroid localization algorithm can solve this problem. Here, the triangle centroid localization algorithm is adopted. In step one, the center point P of the intersecting area should be given:

(8) $$\{\begin{array}{c} x=\frac{P1_{x}+P2_{x}+P3_{x}+P4_{x}}{4}\\ y=\frac{P1_{y}+P2_{y}+P3_{y}+P4_{y}}{4} \end{array}$$

where P1 is the point of intersection between station 3 and station 4, $P1_{x}$ is the value of P1 along the X-axis, and $P1_{y}$ is the value of P1 along the Y-axis.

In step two, the weight derived from difference detection for every side can be calculated as shown in Equation (9). Assume that the levels of difference detection of the four stations are $\Sigma_{1}$, $\Sigma_{2}$, $\Sigma_{3}$ and $\Sigma_{4}$. A low level of difference detection means that the real target’s location is more likely near the station. In reality, the occurrence of intersecting circles with evaluated errors is an event with high probability; thus, the distance decision strategy plays an important role in trilateration. Consequently, we give the weight for every side as follows:

(9) $$\{\begin{array}{c} k_{1}=\frac{\Sigma_{2}+\Sigma_{3}+\Sigma_{4}}{3*(\Sigma_{1}+\Sigma_{2}+\Sigma_{3}+\Sigma_{4})}\\ k_{2}=\frac{\Sigma_{1}+\Sigma_{3}+\Sigma_{4}}{3*(\Sigma_{1}+\Sigma_{2}+\Sigma_{3}+\Sigma_{4})}\\ k_{3}=\frac{\Sigma_{1}+\Sigma_{2}+\Sigma_{4}}{3*(\Sigma_{1}+\Sigma_{2}+\Sigma_{3}+\Sigma_{4})}\\ k_{4}=\frac{\Sigma_{1}+\Sigma_{2}+\Sigma_{3}}{3*(\Sigma_{1}+\Sigma_{2}+\Sigma_{3}+\Sigma_{4})} \end{array}$$

where $k_{1}$, $k_{2}$, $k_{3}$ and $k_{4}$ are the weights for P1, P2, P3 and P4, respectively. A low level of difference detection means a more precise and larger weight. According to the order of weights, we move the center point P to the four intersection points in turn. For example, if $k_{1}$>$k_{2}$>$k_{3}$>$k_{4}$, we move the center point P to $P_{01}$ on the way towards $P_{1}$ at $d_{1}$, then we move point $P_{01}$ to $P_{12}$ on the way towards $P_{2}$ at $d_{2}$, then we move point $P_{12}$ to $P_{23}$ on the way towards $P_{3}$ at $d_{3}$, and then we move point $P_{23}$ to $P_{34}$ on the way towards $P_{4}$ at $d_{4}$. $P_{34}$ is the final location point. In detail, the length of the distance moved is shown in Equation (10):

(10) $$\{\begin{array}{c} d_{1}=\left|\left|P-P_{1}\right|\right|*k_{1}\\ d_{2}=\left|\left|P_{01}-P_{2}\right|\right|*k_{2}\\ d_{3}=\left|\left|P_{12}-P_{3}\right|\right|*k_{3}\\ d_{4}=\left|\left|P_{23}-P_{4}\right|\right|*k_{4} \end{array}$$

## 4. Results

### 4.1. Experimental Setup

MAX Beacon, from a company called Bright Beacon (Chungking, China), iwas chosen as the test beacon. MAX Beacon is equipped with a Nordic51822AA unit, which is a BLE system on a chip. MAX Beacon weighs 65 g and has a lifetime of 2 to 3 years with a broadcast power of −30 dBm to 4 dBm, broadcast frequency of 100 to 1000 ms, and broadcast distance of 3 to 100 m. With a broadcast power of 0 dBm, we can detect the advertising package to as far as 20 m. By adjusting the firmware, we can make MAX Beacon advertise in one channel. We set up a location station with three MAX Beacons. The three MAX Beacons are denoted as MAX beacon1, MAX beacon2 and MAX beacon3. MAX beacon1 broadcasts the message in CH 37, MAX beacon2 broadcasts the message in CH 38 and MAX beacon3 broadcasts the message in CH 39.

**Figure 8:**
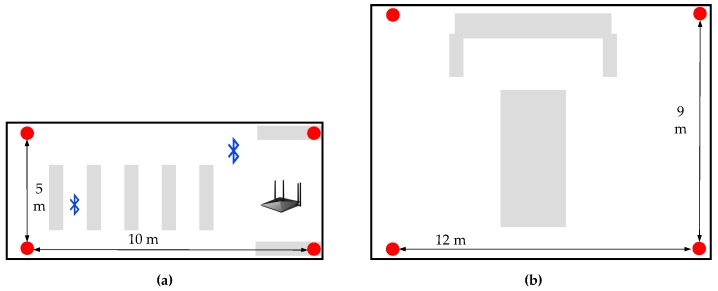
The test setup: (**a**) a classroom (scenario #1); (**b**) an office room (scenario #2). The red point is the location station; the gray bars denote desks and cupboards.

Figure 8 shows two experimental setups. In Figure 8a, four stations are deployed in an office room with a size of 5 m × 10 m. Some furniture (like cupboards, so as to simulate NLOS), WiFi APs and devices embedded with Bluetooth (to simulate 802.11 interruption) are placed in a classroom. Figure 8b is a typical office room, which is similar to Figure 8a but with however a different layout. This office room is in a size of 9 m × 12 m and filled with a big cupboard and a desk. The experimental setup is as real as possible, with some extra artificial interference like moving people and WiFi Aps, to verify the reliability of our proposed methods. It’s true that the practical indoor environment (like a shopping mall) may include several rooms and corridors. However, due to the limitations of BLE technology itself, four station-based BLE trilateration method can only realize accurate positioning inside a room, otherwise the NLOS effect caused by walls can heavily contaminate the RSS measures [32,36].

### 4.2. Performance of the Algorithm

#### 4.2.1. The Distance Decision Strategy

**Figure 9:**
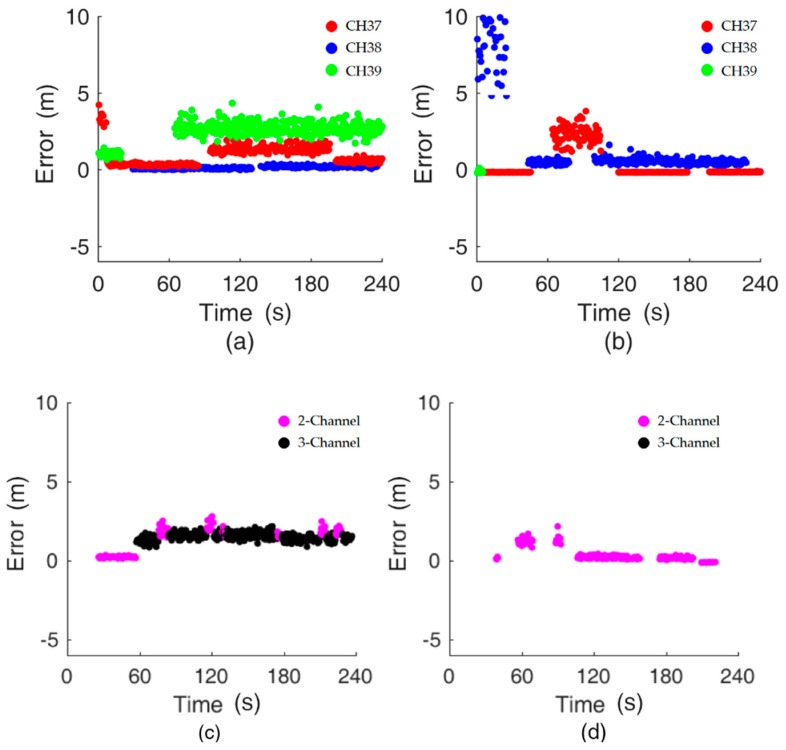
Distance inferred from the information for each channel when using the (**a**) Google Pixel 3 L and (**b**) Huawei P20. Distance value output with the distance decision strategy when using the (**c**) Google Pixel 3 L and (**d**) Huawei P20.

Some of the messages broadcast in the separate channels could be lost. The loss is random and unpredictable, particularly in a complex environment. The typical raw data of the distance decision are illustrated in Figure 9a. Figure 9a demonstrates the typical distribution when we input the median value of the sliding window into the traditional log-distance path loss model to obtain a distance; see Equation (1). The X-axis represents time. From 0 s to 20 s, there are only two valid channels (CH 37 and CH 39). From 30 s to 60 s, the valid channels are CH 37 and CH 38. From 100 s to 180 s, all three channels are available.

As we addressed before, no distance value is output when the number of valid distances is one, which is shown as a gap in Figure 9. The test uses two smartphones (Google Pixel 3 L and Huawei P20) to evaluate the impact of device heterogeneity. For each smartphone, the RSS values of separate channels should be collected in the offline phase at every fixed position of less than 10 m in our study. The range of 10 m is selected because (1) it is enough for our test field and (2) small fluctuations in the RSS will lead to a large change in the estimated distance if a larger range is chosen. Figure 9a,b and Figure 9c,d demonstrate the results of the estimated distance with and without the proposed distance decision strategy.

In Figure 9a,b, without the distance decision strategy, the distance error in separate channels is posted, being as much as ten meters and even more. In contrast, the distance error when using the distance decision strategy, as shown in Figure 9c,d, is less than 2.5 m all the time. About 75% of all scans can output results with Google Pixel 3 L in Figure 9c and around 67% with Huawei P20 in Figure 9d, which shows the effective continuity of proposed methods. Though, the proposed methods will recover from no outputs quickly, see Figure 9d in 60 s. In detail, with consideration of the distance error, Figure 9a,b show better results, implying that both the 2-Channel algorithm and the 3-Channel algorithm can output great results. There is a typical distribution in Figure 9a. We use 3-Channel mostly to determine the distance. Figure 9b not only utilizes 2-Channel but also represents the case of a single channel, which outputs no result. After approximately 170 s in Figure 9a, 2-Channel is adopted instead of 3-Channel, even though three separate RSSs are available, as the case cannot meet the requirement of difference detection of 3-Channel. In other words, 2-Channel and 3-Channel are effective at reducing the distance error to 2 m.

In our experiment, by using separate signal-attenuation models and the distance decision strategy, the complex interference influences the separate channels, which leads to a bad result. To our knowledge, no interference can have the same effect on all separate channels. More carefully, Table 3 shows that for different smartphones, the mean distance error can be less than 2.3 m in such a complicated and changing indoor environment. The mean distance error becomes larger when the real distance increases and smaller when the real distance decreases. The algorithm aims to avoid sudden interference as much as possible rather than facing unpredictable and complicated indoor environments. Therefore, the distance decision strategy possesses the ability to recover from a complicated indoor environment efficiently and effectively.

#### 4.2.2. The Weighted Trilateration

**Figure 10:**
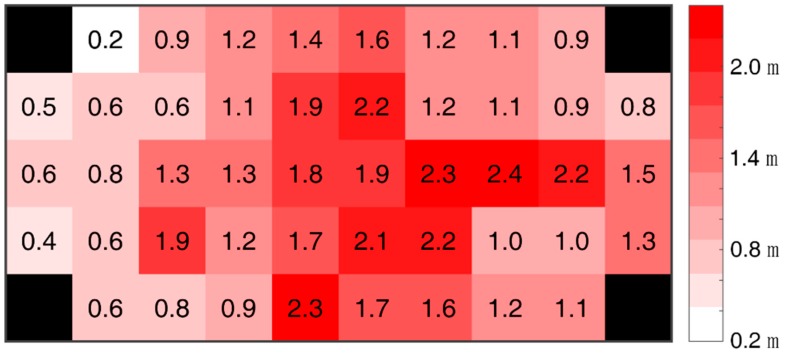
Heatmap of positioning mean absolute error (MAE).

To obtain the performance of weighted trilateration derived from the distance decision strategy, an experimental setup with the Google Pixel 3 L is set up, as shown in Figure 8, with four stations, where 46 points are uniformly distributed. The experiments are carried out by testing at one point (the center of the block) 100 times to calculate the mean absolute error of the positioning results. Each center of the grid in Figure 10 denotes a test position, excluding the black ones, which depict the actual distribution of stations.

In the experiment, the distance between the farthest point and one of the stations is 5 m, and the mean absolute error of the algorithm above is less than 2.4 m at all the points (see Figure 10). This shows the great performance of weighted trilateration according to the decided distance. The mean absolute error decreases when the position is closer to the station and increases when the position is far from the station. Similarly, the error values near the WiFi APs and devices embedded with Bluetooth are larger than those of positions that are far away. The experiment shows that our proposed algorithm could obtain a distance mean error of less than 2.4 m in scenario #1 with the Google Pixel 3 L.

More importantly, a comparison between different algorithms in scenario #1 with the Google Pixel 3 L should be carried out. Table 4 reveals the symbols denoting the different algorithms, and table 5 illustrates the mean absolute error of the different algorithms. Specifically, in a comparison of the precision of the distance errors, aggregate signal-attenuation models with normal distance decision ($A_{1}+B_{1}$), separate signal-attenuation models with normal distance decision ($A_{2}+B_{1}$) or a combination of separate signal-attenuation models and the distance decision strategy ($A_{2}+B_{2}$) is adopted to show the distance error in relation to one of the four stations. Furthermore, in a comparison of the precision of the positioning errors, aggregate signal-attenuation models with the distance decision strategy and traditional trilateration ($A_{1}+B_{1}+C_{1}$) or separate signal-attenuation models with the distance decision strategy and traditional trilateration ($A_{2}+B_{2}+C_{1}$) or weighted trilateration ($A_{2}+B_{2}+C_{2}$) are intensively utilized to analyze the distinct results.

In theory, a wrong distance estimated from any channel will destroy the fusion result in the online phase. The traditional method will adopt all channel information, despite of large error in certain channel. However, the proposed method in this study fully consider the situation of one or two channel’s failure in the distance decision. Additionally, three aggregate distances fused with 2-Channel or 3-Channel ($A_{1}+B_{2}$) is not good as our proposed fusion methods ($A_{2}+B_{2}$) due to that the value of separate channel has the lower variance than value of aggregated channel. Unfortunately, the unstable RSS of aggregate channel make 2-Channel and 3-Channel not working at most of time. All the reason is that the proposed fusion method has a more pure and more stable source data than the aggregated method.

Table 5 shows the different results of the different algorithms. $‘A_{1}+B_{1}$’ cannot obtain a perfect result in our real indoor environment because the distance error can be 8.5 m at 98%. The result of $‘A_{2}+B_{1}’$ could be better, but the MAE is not stable. Furthermore, $‘A_{2}+B_{2}’$ can improve the resulting MAE greatly to 2.0 m at 90%. In view of the positioning error, $‘A_{1}+B_{1}+C_{1}’$ can yield 12.8 m at 98%. For $‘A_{2}+B_{2}+C_{2}’$, we can obtain a better result, that is, 2.2 m at 90%, in contrast to the result of $‘A_{2}+B_{2}+C_{1}’$ with a 90% error, that is, 3.6 m. To test our proposed methods using different smartphones in different scenarios, more experiments should be conducted.

Strictly speaking, the “$A_{1}+B_{1}$” is the aggregated (fusion) methods, and the “$A_{2}+B_{2}$“ is the proposed methods (separate channel plus distance decision). For positioning, the “$A_{1}+B_{1}+C_{1}$” is the most method with traditional trilateration, and the “$A_{2}+B_{2}+C_{1}$” is completely proposed by our study with weighted trilateration.

Here, three experiments are set up: (1) Google Pixel 3 L in scenario #1; (2) Huawei P20 in scenario #1; and (3) Google Pixel 3 L in scenario #2. Experiments (1) and (2) aim to evaluate the effect of different smartphones in the same scenario, while experiments (1) and (3) explore the effect of the same smartphone in different scenarios.

**Figure 11:**
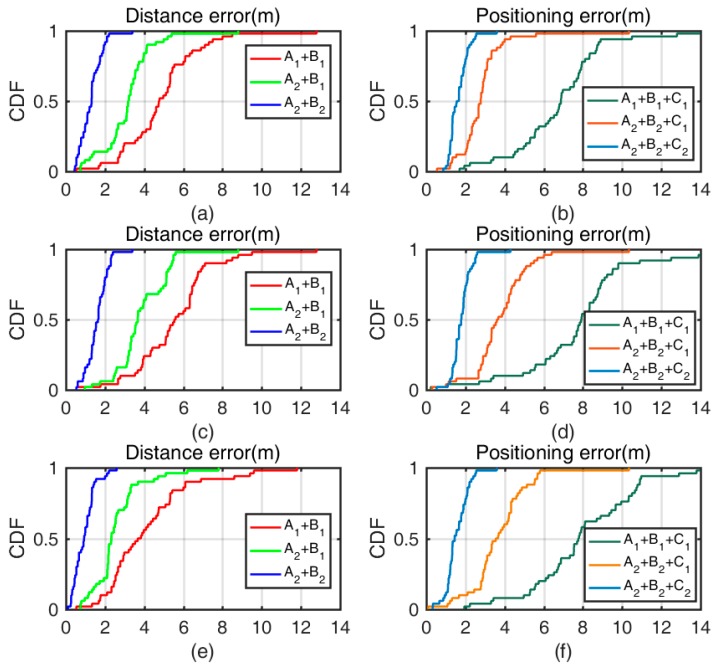
CDF values of the distance error and positioning error when using the (**a,b**) Google Pixel 3 L in scenario #1; (**c,d**) Huawei P20 in scenario #1; and (**e,f**) Google Pixel 3 L in scenario #2.

In experiment (1), the distance error of traditional methods is 8.5 m at 98%, and the result of test methods is 5.2 m at 98%, but they are not stable. Our proposed methods can improve the error to 2.0 m at 90%. In view of the positioning error, traditional methods yields 12.8 m at 98%, and proposed methods is 2.2 m at 90%, in contrast to the result of test methods with a 90% error, namely, 3.6 m; see Figure 11a,b. Errors will change for different smartphones.

In experiment (2), the Huawei P20 is used in scenario #1, and the curve of the CDF is more dispersed than the one in Figure 11a. The distance error of traditional methods is 5.7 m at 50%, and the result of test methods is 3.8 m at 50%. The distance error of traditional methods is 9.1 m at 98%, and the result of test methods is 5.8 m at 98%, both of which are higher values than those in experiment (1) because different smartphones have different responses owing to different chips, electric circuits and materials being used. The result shows that the Google Pixel 3 L has a better response than that of the Huawei P20 in scenario #1. Luckily, our proposed methods can obtain an error of 2.2 m at 90%, which is close to the performance of the Google Pixel 3 L. The result of proposed methods implies that the distance decision strategy can output a good result even when worse results appear for different smartphones. In view of the positioning error, traditional methods result in a value of 14.0 m at 98%, and proposed methods result in a value of 2.4 m at 90%, in contrast to the result of test methods with a 90% error, namely, 4.5 m. The positioning error results add credibility to the idea that our proposed methods can recover from a bad response with smartphones in terms of the distance and positioning errors; see Figure 11c,d. Experiments (1) and (2) show the reliability in the heterogeneity of smartphones.

In experiment (3), the Google Pixel 3 L is used in scenario #2. Fewer WiFi APs or devices embedded with Bluetooth but more space are used in scenario #2, which depicts an office room. The distance error of traditional methods is 8.5 m at 98%, and the result of test methods is 5.1 m at 98%, both of which are smaller than those in experiment (1) because the influence of some devices running in 802.11 [21] is smaller. The result shows that scenario #2 is purer than scenario #1. Of course, the performance of proposed methods can be improved because the distance error is calculated in relation to one station, whose distance error is 2.1 m at 98%. Different scenarios yield different results. In view of the positioning error, traditional methods yields a value of 13.9 m at 98%. Test methods yield a value of 3.9 m at 90%, and proposed methods, a value of 2.4 m at 90%. The positioning error is slightly larger than that in scenario #1. In the experimental setup, the distance error is larger when the smartphone is far from the station, which affects the positioning error. Additionally, our proposed methods can recover in different scenarios (see Figure 11c,d). It is apparent that a more precise result can be obtained when more location stations are deployed, but this will lead to higher cost and complexity. Experiments (1) and (3) show the reliability in the heterogeneity of environment.

In summary, Figure 11 demonstrates the CDF (cumulative distribution function) of different algorithms for different smartphones in different scenarios. The proposed methods (separate channel plus distance decision) is better than the aggregated (fusion) methods in estimating distance. Even different smartphones have different responses: The proposed methods can output a distance with less error. Similarly, the proposed methods with weighted trilateration can output a stable and precise position in different scenarios, making it better than the most methods with traditional trilateration. The advantage of our proposed methods is demonstrated above in the stability of data source, the robustness against complex indoor environment, the effective continuity of outputting results, the powerful recovery from inactive state, the reliability in the heterogeneity of smartphones environments.

## 5. Conclusions and Future Work

This paper proposed an adaptable and robust algorithm based on separate channels, separate signal-attenuation models, the distance decision strategy and weighted trilateration using BLE beacons with off-the-shelf smartphones. The experimental results show that the proposed methods (separate channel plus distance decision) for obtaining the distance accuracy yields values less than 2.2 m at 90%; moreover, the result of the aggregated (fusion) methods was 7.1 m for different smartphones. In addition, the proposed methods with weighted trilateration for achieved the positioning accuracy of less than 2.4 m at 90% from 3.6 m away with the most methods with traditional trilateration. The positioning error was reduced by 33% to 38% for different building environments. Based on the test results, we conclude that the method is robust and precise for indoor positioning with BLE beacons and smartphones.

In the future, much of this work could be improved. The system should be improved to a decimeter positioning system that can handle greater demands and more stations, resulting in more accuracy. First, the positioning errors of different deployments of stations should be investigated. Second, complicated interference, such as interruption at the same radio frequency, should be explored and at least minimized. The measurement of interference can provide a reference for reducing any negative influence on indoor positioning. Last, the new standard of Bluetooth 5.1 can supply a method for measuring the AOA and AOD with off-the-shelf products rather than simply simulating data [19]. Combining the information from separate channels with AOA and AOD should be considered. Above all, a more precise, robust, cheap, easily deployed, popular and well-maintained system for indoor positioning should be put forward.

## Figures and Tables

**Table 1:** Distance errors in CH 37, CH 38 and CH 39.

Channel ID	30% of Time in Error (m)	60% of Time in Error (m)	90% of Time in Error (m)
CH 37	2.0	3.5	5.2
CH 38	1.6	2.6	4.6
CH 39	1.1	1.9	4.0

**Table 2:** Results of the test for different cases.

Case	A	B	C
Number of Tests	10	10	10
Number of Empty Outputs	1	6	10

**Table 3:** The mean distance error with increasing distance for different phones.

	Dist. (m)	0.2	1.4	2.6	3.8	5.0	6.2	7.4	8.6	9.8
Error (m)										
Google Pixel 3 L		0.1	0.2	0.8	1.1	1.2	1.5	1.2	1.9	2.0
Huawei P20		0.1	0.4	0.1	1.4	1.3	1.5	1.3	1.8	2.3

**Table 4:** Symbols denoting different algorithms.

Algorithm	Symbol
Aggregate Channel	$A_{1}$
Separate Channel	$A_{2}$
Normal Distance Decision	$B_{1}$
Proposed Distance Decision	$B_{2}$
Traditional Trilateration	$C_{1}$
Weighted Trilateration	$C_{2}$

**Table 5:** Comparison of the precision of the mean absolute error (m).

Mean Absolute Error	Algorithm	90%	98%
Distance Error	$A_{1}+B_{1}$(Tradition)	7.1	8.5
	$A_{2}+B_{1}$(Test)	4.6	5.2
	$A_{2}+B_{2}$(Proposed)	2.0	2.2
Positioning Error	$A_{1}+B_{1}+C_{1}$(Tradition)	8.8	12.8
	$A_{2}+B_{2}+C_{1}$(Test)	3.6	4.3
	$A_{2}+B_{2}+C_{2}$(Proposed)	2.2	2.5
